# Association Between Mode of Delivery and Postpartum Depression: The Japan Environment and Children’s Study (JECS)

**DOI:** 10.2188/jea.JE20210117

**Published:** 2023-05-05

**Authors:** Sachiko Baba, Satoyo Ikehara, Ehab S. Eshak, Kimiko Ueda, Tadashi Kimura, Hiroyasu Iso

**Affiliations:** 1 Bioethics and Public Policy, Department of Social Medicine, Osaka University Graduate School of Medicine, Osaka, Japan; 2 Public Health, Department of Social Medicine, Osaka University Graduate School of Medicine, Osaka, Japan; 3 Public Health, Faculty of Medicine, Minia University, Minia, Egypt; 4 Osaka Women’s and Children’s Hospital, Osaka, Japan; 5 Obstetrics and Gynecology, Osaka University Graduate School of Medicine, Osaka, Japan

**Keywords:** cesarean section, postpartum depression, psychological distress, breastfeeding, prospective study

## Abstract

**Background:**

Postpartum depression (PPD) has been associated with adverse health outcomes, including maternal suicide. Mode of delivery has been suggested to be a risk factor for PPD, but no large cohort study has examined the association between mode of delivery and PPD. We aimed to examine the association between mode of delivery and risks of PPD at 1 and 6 months after childbirth.

**Methods:**

In a nationwide study of 89,954 mothers with a live singleton birth, we examined the association between mode of delivery and risks of PPD. PPD was evaluated using the Edinburgh Postnatal Depression Scale (≥13) at 1 and 6 months after childbirth. Odds ratios (ORs) with 95% confidence intervals (CIs) of PPD were calculated using multivariable logistic regression analyses after adjustment of antenatal physical, socioeconomic, and mental factors.

**Results:**

Among 89,954 women, 3.7% and 2.8% had PPD at 1 and 6 months after childbirth, respectively. Compared with unassisted vaginal delivery, cesarean section (CS) was marginally associated with PPD at 1 month but not at 6 months; adjusted ORs were 1.10 (95% CI, 1.00–1.21) and 1.01 (95% CI, 0.90–1.13), respectively. The association with PPD at 1 month was evident in women with antenatal psychological distress (adjusted OR 1.15; 95% CI, 1.03–1.28). The observed associations were attenuated after adjusting for infant feeding method.

**Conclusion:**

Women who had antenatal psychological distress and underwent CS delivery may be regarded as a target for monitoring PPD.

## INTRODUCTION

Postpartum depression (PPD) is a major concern in reproductive health.^1^ Ten to twenty percent of women suffer from depression after childbirth, both worldwide and in Japan.^1^^–^^4^ PPD has shown to be associated with adverse health outcomes, such as maternal suicidal ideation and impaired maternal-infant interaction, even soon after delivery, and with children’s emotional, cognitive, and behavioral problems over the long term.^5^^–^^8^ The most serious adverse health outcome is suicide in postpartum mothers. In Japan, maternal and late-maternal deaths by suicide were reported to be 8.7 per 100,000 births, with 63 cases (23 during pregnancy and 40 within a year after delivery) from 2005 to 2014 in Tokyo, Japan; this is in contrast to the nationwide low maternal mortality rate (ie, 2.7 per 100,000 births in 2014), which is thought to be due to maternal physical complications during pregnancy.^9^ Globally, postpartum suicide has been listed as a possible cause of pregnancy-related death in the 10^th^ revision of the International Classification of Diseases and Related Health Problems (ICD-10).^10^ Therefore, the prevention of PPD is important in terms of preventing avoidable maternal deaths. Reported antenatal risk factors for PPD range from physical, socioeconomic, and mental factors.^1^^,^^3^^,^^10^^–^^13^

Mode of delivery has been also suggested to be a risk factor for PPD.^12^^,^^14^ In a meta-analysis of 28 studies, cesarean section (CS) was associated with increased risk of PPD.^15^ This meta-analysis included five Asian studies; three of them had small cohort sample sizes of less than 600, and two were cross-sectional studies. A Canadian cohort study of 2,560 women showed no association between CS and PPD among the overall sample of women; however, in interaction models, CS was associated with an increased risk of PPD in Canadian-born women and a decreased risk in non-Canadian-born women, suggesting a difference in the subjective “desired” mode of delivery according to culture and ethnicity.^16^ Because the rate of CS in Japan is on the rise,^17^ it is important to examine the potential adverse outcomes of CS. A previous study of 479 Japanese women showed that emergency CS was associated with an increased risk of PPD, but the number of CS cases was only 109 and the study subjects were limited to primiparous women aged 35 years and older.^18^ Other than CS, a previous study involving 707,701 Swedish women showed that instrumental vaginal delivery was associated with PPD within a year after childbirth.^19^ Because vaginal delivery comprises 80% of all deliveries in Japan,^17^ it is important to examine the effect of assisted and instrumental vaginal deliveries in reference to unassisted (normal) vaginal delivery on PPD among Japanese women if the data allows. One previous study examined the association of induced vaginal delivery and CS with neonatal outcomes compared with unassisted vaginal delivery,^20^ but there has been no study that examined the association between these modes of delivery and PPD.

The Japan Environment and Children’s Study (JECS) collected information about the mode of delivery and PPD among large nationwide population samples. Using these data, we assessed the association between mode of delivery (unassisted vaginal delivery, assisted vaginal delivery, instrumental delivery, and CS) and risk of PPD at 1 and 6 months after childbirth.

## METHODS

### Study population

The JECS is a nationwide government-funded birth cohort study that began recruiting expectant mothers in January 2011 and finished recruiting in March 2014. Fifteen Regional Centres (Hokkaido, Miyagi, Fukushima, Chiba, Kanagawa, Koshin, Toyama, Aichi, Kyoto, Osaka, Hyogo, Tottori, Kochi, Fukuoka, and South Kyushu/Okinawa) were selected. Each Centre recruited women during early pregnancy at obstetrics clinics/hospitals, and local municipal offices issuing the Maternal and Child Health Handbook. The JECS Study Protocol and details have been published elsewhere.^21^^–^^23^ The present study is based on the jecs-ag-20160424 dataset, which was released in June 2016 and revised in October 2016.

### Participants

Among 104,065 fetal records, we only included mothers with a live singleton infant (*n* = 92,790). Complete information on mode of delivery was available for 92,356 mothers. In addition to mothers with complete information on their Edinburgh Postnatal Depression Scale (EPDS) scores, mothers with total EPDS scores greater than the cut-off score, even with missing values, were included and regarded as positive for PPD. In contrast, mothers with total EPDS scores less than the cut-off score, when estimated scores for missing items did not exceed 3 points, were included and regarded as negative for PPD. Thus, PPD status at 1 month and 6 months postpartum was available for 89,954 and 84,858 mothers out of the 92,790 mothers (96.9% and 91.5%), respectively.

### Ethical issues

The JECS protocol was reviewed and approved by the Ministry of the Environment’s Institutional Review Board on Epidemiological Studies and by the Ethics Committees of all participating institutions. Written informed consent was obtained from all participants. The JECS was conducted in accordance with the Declaration of Helsinki and other nationally valid regulations and guidelines.

### Variables

Self-administered questionnaires were conducted during the first, second, and third trimesters, and at 1 and 6 months after childbirth. Information obtained included demographic characteristics, medical history, and socioeconomic and mental factors. Maternal anthropometric data before pregnancy, as well as data on complications during pregnancy, medical history, and perinatal outcomes, such as mode of delivery and gestational age at birth, were collected through data transcription from medical records. Data transcription was performed by physicians, nurses, midwives, or research coordinators.

Mode of delivery was categorized as unassisted vaginal delivery, assisted vaginal delivery, instrumental vaginal delivery, and CS. Assisted vaginal delivery meant vaginal delivery with labor induction, and instrumental vaginal delivery meant vaginal delivery with the use of either vacuum or forceps extraction. CS included both elective and emergency CS in the questionnaires, so we could not distinguish one from the other in our sample.

We collected Japanese-language EPDS scores at 1 and 6 months after childbirth. The total score of the EPDS, which comprises 10 items listed on a four-point Likert scale from 0 to 3 according to the increasing severity of symptoms, ranges from 0 to 30.^24^ Okano et al translated the EPDS into Japanese, back-translated the scale, conducted the test-retest method to examine the reliability, and calculated Cronbach’s α (0.78).^25^ The cut-off score to indicate PPD in the EPDS varied in previous literature. According to a systematic review of 37 studies (six studies using the English version of the EPDS, 25 studies using a non-English version of the EPDS, and two studies using English and non-English versions of the EPDS), the cut-off score of 9/10 was suggested to indicate possible PPD and 12/13 was suggested to indicate probable PPD.^26^ We used the cut-off score of 12/13 to signify PPD in this study, which could allow international comparisons with previous studies.^15^^,^^26^ The covariates based on prior studies included women’s age, parity, pre-pregnancy body mass index (BMI), weight gain during pregnancy, gestational week at birth, and newborn complications at birth as physical factors; marital status, education, annual family income, and residential area as socioeconomic factors; antenatal mental status and history of mental diseases as mental factors; and infant feeding method.

Women’s age was categorized as 5-year age groups, and parity as primiparous or multiparous. Pre-pregnancy BMI was calculated as the weight in kilograms divided by the square of the height in meters and re-categorized into underweight, normal, and overweight and obese according to the World Health Organization classification^27^ as <18.50, 18.50–24.99, and ≥25.00 kg/m^2^, respectively. Weight gain during pregnancy was calculated by subtracting weight just before delivery from pregestational weight and re-categorized into quantiles. Gestational week at birth was dichotomously categorized into term (≥37 gestational weeks) or preterm (≤36 gestational weeks). Newborn complications at birth were dichotomously categorized into yes or no. Marital status was categorized as married, never married, divorced, or widowed. Education was categorized as junior high school, high school, vocational school, junior college, or university and higher. Equivalent family income was calculated by dividing the average amount in a six-scale family income category by the square root of the number of family members and re-categorized into quantiles. Women’s antenatal mental status was assessed using the Japanese version of the Kessler 6 (K6) scale. The K6 score was originally used for screening “serious mental illness”^28^ or mood and anxiety disorder (ie, psychological distress)^29^ in the general population. The optimal cut-off score for screening mood and anxiety disorders, or possible psychological distress in the general population was 4/5.^30^^,^^31^ Therefore, we dichotomously categorized women into those with (≥5) or those without (0–4) antenatal psychological distress. History of mental diseases was determined using a question regarding history of diagnosed mental diseases (depression, autonomic imbalance, anxiety disorder, and schizophrenia) in the past. Infant feeding method at 1 month was categorized as exclusive breastfeeding, bottle feeding, or mixed. Residential areas were represented by the locations of the 15 Regional Centres where the data were collected.

### Statistical analyses

The categories’ proportions of each variable in the total sample were calculated and tested using a chi-square test across each mode of delivery. Multivariable logistic regression analyses were used to estimate the associations between mode of delivery and PPD. Odds ratios (ORs) and 95% confidence intervals (CIs) were calculated after adjusting for potential confounders. Using unassisted vaginal delivery as the reference group, we estimated the risk of PPD (EPDS score ≥13 at 1 and 6 months after childbirth) among women whose delivery was assisted, instrumental, or CS. In model 1, we adjusted for the women’s age. In model 2, we further adjusted for parity, pre-pregnancy BMI, weight gain during pregnancy, gestational week at birth, and newborn complications at birth. In model 3, we further adjusted for marital status, education, annual family income, mental status, history of antidepressant use, and residential area. We further adjusted for infant feeding method in an additional analysis, and conducted a stratified analysis (*n* = 89,669) using the method of infant feeding to check for any effect modification in the associations between mode of delivery and risk of PPD by infant feeding method. In addition, a stratified analysis by antenatal mental status was conducted for those whose antenatal mental status was obtained (*n* = 88,432). All analyses were performed using Statistical Analysis Software version 9.4 (SAS Institute, Inc., Cary, NC, USA).

## RESULTS

Table 1 shows the baseline characteristics of 89,954 women according to mode of delivery. Among them, 3,546 (3.7%) and 2,584 (2.8%) women had PPD at 1 and 6 months after childbirth, respectively. Among all women, 51,507 (57.2%) had an unassisted vaginal delivery, and 16,234 (18.0%) needed an assisted vaginal delivery, 5,381 (6.0%) had an instrumental delivery, and 16,802 (18.7%) had CS. Among instrumental deliveries, 5,131 (96.2%) were vacuum deliveries, and the rest were forceps deliveries (data not shown). Women who gave birth by CS were older, primiparous, obese, preterm, and had the lowest weight gain during pregnancy, newborn complications at birth, higher income, lower antenatal mental status, and used bottle or mixed feeding with their infants.

**Table 1.** Characteristics of participants

Characteristics		Total (*n* = 89,954)	Unassisted vaginal delivery (*n* = 51,507)	Assisted vaginal delivery (*n* = 16,234)	Instrumental delivery (*n* = 5,381)	Cesarean Section (*n* = 16,802)	
		*n*	(%)	(%)	(%)	(%)	*P*-value
Postpartum depression (EPDS 13 and over)	At 1 month after childbirth	3,546	3.7	4.1	4.4	4.5	<0.0001
	At 6 months after childbirth	2,584	2.8	2.9	3.1	3.0	0.72

Age, years	<20	769	1.0	0.8	0.8	0.6	<0.0001
	20–29	32,707	38.7	37.3	39.5	27.2	
	30–39	52,305	56.7	57.4	55.7	64.1	
	≥40	4,173	3.6	4.5	4.1	8.1	

Parity	Primiparous	37,314	35.0	53.1	68.9	41.5	<0.0001

Prepregnancy BMI, kg/m^2^	Underweight	14,525	17.7	14.6	17.5	12.6	<0.0001
	Normal	65,862	74.0	73.1	73.9	70.7	
	Obese	9,498	8.3	12.3	8.5	16.6	
	Missing	69	0.1	0.1	0.0	0.1	

Weight gain during pregnancy, kg	Lowest (<8.0)	21,667	23.6	22.1	22.2	28.1	<0.0001
	Second lowest (8.0–10.29)	23,059	26.4	24.4	24.3	25.0	
	Second highest (10.30–12.69)	21,047	23.8	23.5	24.4	21.6	
	Highest (>12.70)	22,385	24.1	28.7	27.1	23.0	
	Missing	1,796	2.1	1.4	2.0	2.3	

Gestational age	Preterm	4,025	3.4	2.5	2.2	10.4	<0.0001

Newborn’s complication	Yes	8,696	7.0	9.5	9.3	18.2	<0.0001

Marital status	Married	84,979	94.6	93.9	93.6	94.9	<0.0001
	Never (not yet)	3,211	3.4	4.0	4.9	3.1	
	Divorced	736	0.8	0.7	0.6	1.0	
	Widowed	15	0.0	0.0	0.1	0.0	
	Missing	1,013	1.2	1.3	0.7	1.0	

Education	Junior high school	4,109	4.7	4.6	3.6	4.3	<0.0001
	High school	27,767	30.8	30.2	31.8	31.4	
	Vocational school	21,765	23.9	24.6	25.8	24.3	
	Junior college	15,715	17.4	17.4	16.5	18.1	
	University and higher	19,382	21.9	22.1	21.3	20.2	
	Missing	1,216	1.4	1.2	1.0	1.6	

Family equivalent income, million JPY/year	Lowest (<173.2)	23,393	27.9	22.4	19.9	25.5	<0.0001
	Second lowest (173.2–250)	12,997	13.2	17.2	18.7	14.2	
	Second highest (250–353.6)	27,460	30.7	30.4	29.8	30.4	
	Highest (>353.6)	18,392	19.5	21.8	22.6	21.5	
	Missing	7,712	8.7	8.3	9.0	8.4	

Antenatal mental status	Possible psychological distress (K6 ≥5)	25,548	28.2	28.4	30.1	28.4	0.0002

History of mental diseases	Yes	4,570	4.8	5.5	4.8	5.5	<0.0001

Infant feeding method	Bottle or mixed feeding	51,961	53.9	60.2	67.6	64.0	<0.0001

BMI, body mass index; EPDS, Edinburgh Postnatal Depression Scale; JPY, Japanese yen.

Compared with women who gave birth by unassisted vaginal delivery, women who gave birth by assisted vaginal delivery, instrumental delivery, or CS had an increased risk of PPD at 1 month in the age-adjusted analysis: ORs were 1.12 (95% CI, 1.02–1.23), 1.20 (95% CI, 1.05–1.38), and 1.30 (95% CI, 1.19–1.42), respectively (Table 2, model 1). In the multivariable-adjusted models, the increased risk of PPD at 1 month after CS delivery was attenuated: adjusted OR was 1.13 (95% CI, 1.04–1.24) after controlling for physical factors (model 2), and the association was marginal after further controlling for socioeconomic and antenatal mental factors (model 3): adjusted OR was 1.10 (95% CI, 1.00–1.21). In the additional analysis where we further controlled for infant feeding method, the association did not reach statistical significance: the adjusted OR was 1.07 (95% CI, 0.97–1.17) (*P* = 0.20).

**Table 2.** Multivariable odds ratios (ORs) of postpartum depression at 1 or 6 months after birth according to mode of delivery

	Model1^a^ OR (95% CI)	Model2^b^ OR (95% CI)	Model3^c^ OR (95% CI)	Additional analysis^d^ OR (95% CI)
At 1 month				
Unassisted vaginal delivery	Ref	Ref	Ref	Ref
Assisted vaginal delivery	1.12 (1.02–1.23)	1.01 (0.92–1.11)	1.05 (0.95–1.16)	1.03 (0.93–1.14)
Instrumental	1.20 (1.05–1.38)	1.04 (0.91–1.20)	1.03 (0.89–1.19)	1.00 (0.86–1.16)
Cesarean section	1.30 (1.19–1.42)	1.13 (1.04–1.24)	1.10 (1.00–1.21)	1.07 (0.97–1.17)
At 6 months				
Unassisted vaginal delivery	Ref	Ref	Ref	Ref
Assisted vaginal delivery	1.05 (0.94–1.17)	1.04 (0.93–1.15)	1.07 (0.95–1.20)	1.06 (0.94–1.19)
Instrumental	1.11 (0.94–1.31)	1.13 (0.96–1.33)	1.15 (0.96–1.37)	1.14 (0.95–1.35)
Cesarean section	1.14 (1.02–1.26)	1.07 (0.96–1.19)	1.01 (0.90–1.13)	1.00 (0.89–1.12)

CI, confidence interval; OR, odds ratio.

^a^Adjusted for age.

^b^Adjusted further for parity, prepregnancy BMI, weight gain during pregnancy, gestational age at birth, and newborn’s complication at birth.

^c^Adjusted further for marital status, education, family income, antenatal mental status, history mental diseases, and residential area.

^d^Adjusted further for infant feeding method.

At 6 months from childbirth, women with instrumental delivery showed an increased, though insignificant, risk of PPD: OR was 1.15 (0.96–1.37) (*P* = 0.12) in model 3. Women who had assisted vaginal delivery or CS showed no increased risk of PPD in multivariable logistic regression models.

Table 3 shows the multivariable ORs of PPD at 1 and 6 months according to mode of delivery, stratified by antenatal mental status. Among women with antenatal psychological distress, those who gave birth by CS had a significantly higher risk of PPD at 1 month compared with women who gave birth by unassisted vaginal delivery: OR was 1.15 (95% CI, 1.03–1.28) after adjusting for all antenatal factors (model 3). The risk was attenuated as 1.11 (95% CI, 0.99–1.24) (*P* = 0.07) in the additional analysis that was further adjusted for infant feeding method. Among women with no antenatal psychological distress, there was no association between mode of delivery and PPD at 1 month. At 6 months, there was no association between mode of delivery and PPD in either strata of the antenatal mental status.

**Table 3.** Multivariable odds ratios (ORs) of postpartum depression at 1 or 6 months after birth according to mode of delivery, stratified by antenatal mental status

	Number at risk	Case	Model1^a^ OR (95% CI)	Model2^b^ OR (95% CI)	Model3^c^ OR (95% CI)	Additional analysis^d^ OR (95% CI)
At 1 month						
No psychological distress (*n* = 62,891)						
Unassisted vaginal delivery	36,110	393	Ref	Ref	Ref	Ref
Assisted vaginal delivery	11,389	159	1.29 (1.07–1.55)	1.07 (0.88–1.29)	1.14 (0.94–1.38)	1.11 (0.91–1.34)
Instrumental	3,689	60	1.50 (1.14–1.97)	1.13 (0.86–1.49)	1.12 (0.85–1.48)	1.07 (0.81–1.41)
Cesarean section	11,703	169	1.38 (1.15–1.65)	1.14 (0.94–1.38)	1.15 (0.96–1.40)	1.11 (0.92–1.34)
Psychological distress (*n* = 25,541)						
Unassisted vaginal delivery	14,541	1,442	Ref	Ref	Ref	Ref
Assisted vaginal delivery	4,604	487	1.08 (0.97–1.21)	1.00 (0.90–1.12)	1.03 (0.92–1.15)	1.01 (0.90–1.13)
Instrumental	1,621	171	1.07 (0.91–1.27)	0.97 (0.82–1.15)	0.98 (0.82–1.16)	0.96 (0.80–1.14)
Cesarean section	4,782	570	1.26 (1.13–1.40)	1.14 (1.02–1.27)	1.15 (1.03–1.28)	1.11 (0.99–1.24)
At 6 months						
No psychological distress (*n* = 59,901)						
Unassisted vaginal delivery	34,412	291	Ref	Ref	Ref	Ref
Assisted vaginal delivery	10,835	109	1.20 (0.96–1.50)	1.18 (0.94–1.48)	1.21 (0.96–1.52)	1.19 (0.95–1.50)
Instrumental	3,564	38	1.26 (0.90–1.78)	1.27 (0.90–1.79)	1.31 (0.92–1.86)	1.29 (0.91–1.82)
Cesarean section	11,090	107	1.19 (0.95–1.49)	1.17 (0.93–1.47)	1.15 (0.91–1.44)	1.12 (0.89–1.42)
Psychological distress (*n* = 23,732)						
Unassisted vaginal delivery	13,509	1,117	Ref	Ref	Ref	Ref
Assisted vaginal delivery	4,294	357	1.02 (0.90–1.15)	1.01 (0.89–1.14)	1.02 (0.90–1.17)	1.01 (0.89–1.16)
Instrumental	1,528	130	1.03 (0.85–1.25)	1.06 (0.87–1.28)	1.08 (0.89–1.32)	1.07 (0.88–1.31)
Cesarean section	4,401	376	1.07 (0.95–1.21)	1.01 (0.89–1.14)	1.00 (0.88–1.14)	0.99 (0.87–1.12)

CI, confidence interval; OR, odds ratio.

^a^Adjusted for age.

^b^Adjusted further for parity, prepregnancy BMI, weight gain during pregnancy, gestational age at birth, and newborn’s complications at birth.

^c^Adjusted further for marital status, education, family income, history of mental diseases, and residential area.

^d^Adjusted further for infant feeding method.

Table 4 shows the multivariable ORs of PPD at 1 and 6 months according to mode of delivery, stratified by infant feeding method. The *P* for interactions between mode of delivery and infant feeding method towards the risk of PPD at both 1 and 6 months were >0.1. At 1 month from childbirth among those who used a bottle or mixed feeding, CS tended to be associated with increased risk of PPD compared with unassisted vaginal delivery, but the association was attenuated: adjusted OR was 1.10 (95% CI, 0.99–1.23) (*P* = 0.09). At 6 months from childbirth among those who used a bottle or mixed feeding, the increased risk of PPD with instrumental delivery was augmented and reached the level of significance: adjusted OR was 1.25 (95% CI, 1.02–1.52).

**Table 4.** Multivariable odds ratios of postpartum depression at 1 and 6 months after childbirth according to mode of delivery, stratified by infant feeding method

	Number at risk	Case	OR^a^ (95% CI)
At 1 month			
Exclusive breastfeeding (*n* = 37,708)			
Unassisted vaginal delivery	23,598	557	Ref
Assisted vaginal delivery	6,414	154	1.02 (0.84–1.24)
Instrumental	1,732	47	1.06 (0.77–1.46)
Cesarean section	5,964	153	0.99 (0.82–1.21)
Bottle or mixed feeding (*n* = 51,961)			
Unassisted vaginal delivery	27,796	1,328	Ref
Assisted vaginal delivery	9,778	506	1.05 (0.94–1.18)
Instrumental	3,638	189	1.00 (0.85–1.19)
Cesarean section	10,749	596	1.10 (0.99–1.23)
*P for interaction*			0.60
At 6 months			
Exclusive breastfeeding (*n* = 35,825)			
Unassisted vaginal delivery	22,461	546	Ref
Assisted vaginal delivery	6,080	145	1.05 (0.86–1.28)
Instrumental	1,662	34	0.87 (0.60–1.26)
Cesarean section	5,622	133	0.90 (0.73–1.11)
Bottle or mixed feeding (*n* = 48,770)			
Unassisted vaginal delivery	26,049	896	Ref
Assisted vaginal delivery	9,201	327	1.07 (0.93–1.24)
Instrumental	3,477	135	1.25 (1.02–1.52)
Cesarean section	10,043	362	1.04 (0.91–1.20)
*P for interaction*			0.30

CI, confidence interval; OR, odds ratio.

^a^Adjusted for age, parity, prepregnancy BMI, weight gain during pregnancy, gestational age at birth, newborn’s complication at birth, marital status, education, family income, antenatal mental status, history of mental diseases, and residential area.

## DISCUSSION

In this nationwide cohort study, we found that CS was marginally associated with an increased risk of PPD at 1 month, but not at 6 months after childbirth after adjustment for antenatal physical, socioeconomic, and mental factors. This tendency was attenuated after adjusting for infant feeding method. In the stratified analyses, we found that an association between CS and risk of PPD at 1 month was evident among women with psychological distress.

Our result indicating a marginal association between CS and PPD at 1 month was consistent with the findings from previous studies that did not adjust for infant feeding method; among 511,422 Finnish women (OR 1.23; 95% CI, 1.06–1.43),^32^ and among 17,648 American women (OR 1.3; 95% CI, 1.1–1.5).^33^ Yet, a Chinese study of 1,823 women and a Japanese study of 99,202 women showed no association between mode of delivery and risk of PPD at 4 weeks, with respective ORs of 1.13 (95% CI, 0.92–1.22),^34^ and 1.02 (95% CI, 0.97–1.08),^35^ partly due to different outcome settings. On the other hand, the null association between CS and risk of PPD at 6 months from childbirth found in our study was similar to the findings from previous studies with 55,814 Norwegian women,^36^ 1,379 Qatari women,^37^ and 99,202 Japanese women.^35^

The mechanism by which CS may influence risk of PPD is not clear.^1^^,^^3^ Biological mechanisms, such as hormonal changes, may play a role in the development of PPD.^1^ The levels of steroid hormones, including estradiol, progesterone, and cortisol, are known to dramatically decrease after childbirth regardless of mode of delivery.^1^ However, there has been debate concerning the role of steroid hormones in depression in general and in PPD.^38^

Also, maternal oxytocin concentrations were associated with the risk of PPD.^39^ The oxytocin level is known to sharply increase during vaginal delivery,^40^ and the mean plasma oxytocin level was significantly higher in unassisted vaginal labor (30.6; standard deviation, 5.0 µU/mL) compared with that in elective CS (22.6; standard deviation, 1.4 µU/mL).^41^ However, a recent systematic review indicated that the infusion of synthetic oxytocin for elective CS raised oxytocin to levels equivalent to those during unassisted vaginal labor.^40^ Further, the oxytocin level after childbirth is substantially affected by lactation, and lactation increased the oxytocin level and the number of oxytocin pulses by lactation was higher after unassisted vaginal labor than that after CS.^42^ Thus, the mechanism by which CS biologically affects risk of PPD remains largely unexplained. Unfortunately, we did not collect information about hormone levels in this study. Further studies are necessary to examine the role of these hormones in the association between mode of delivery and PPD.

There are possible psychological mechanisms which include various kinds of stresses derived from CS that could induce PPD. Generally, stressful life events are known to lead to PPD.^1^^,^^3^ With CS, the inflammation caused by the surgical operation and trauma,^43^^,^^44^ possible local temporary infection,^43^ and chronic pelvic pain^45^ may increase mental and physical stresses.^11^^,^^15^^,^^46^ In addition, having CS may be associated with feelings of failure,^46^ lack of control,^46^ fear of birth,^32^ reduced self-esteem,^46^ and perception of negative delivery experience,^47^ all of which could contribute to the higher risk of PPD. Moreover, societal and women’s longstanding belief that “normal birth” is a “vaginal birth”^16^ may affect the mental status of women who undergo CS (ie, not a “normal birth”). This belief does not seem to be prevalent in Canada,^16^ where the CS rate has been more than 40%; the CS rate was comparatively lower (19%) in our study population.

In our study, the association between CS and PPD at 1 month after childbirth was confined to women with antenatal psychological distress. Women with antenatal psychological distress may be more vulnerable to the stresses and fears discussed above. Therefore, more antenatal psychological support should be offered to pregnant women with antenatal psychological distress to reduce the possibility of CS.

Evidence for the association between breastfeeding and PPD is inconclusive so far. A bidirectional association was recently suggested, meaning that failure of breastfeeding, or choosing not to engage in breastfeeding, may increase the risk of PPD, while PPD itself may reduce the ability and motivation to breastfeed the newborn.^48^ In our study, the method of infant feeding seems to be a strong confounding factor because the association between CS and PPD in the main analysis was greatly attenuated by adjusting for the infant feeding method in the additional analysis. However, we obtained an insignificant interaction by infant feeding method in the stratified analysis (*P* = 0.60). The association between instrumental delivery PPD was unexpectedly evident at 6 months among women who did not use exclusive breastfeeding. Further research is necessary to confirm or reject the causal effect of breastfeeding on PPD, as well as its moderator effect on the association between different modes of delivery and PPD.

The major strengths of this study include the use of data from a nationwide cohort study with a large sample size and ample information. We adjusted for potential confounding variables, such as maternal physical, socioeconomic, and antenatal mental factors, and also conducted stratified analyses. We set two time points for the outcomes, at 1 and 6 months after childbirth, which enabled us to clarify how long the effect of delivery mode on risk of PPD lasted.

However, several limitations should also be addressed. First, there was no information to distinguish between elective and emergency CS. According to the national database of health insurance claims, in 2013, emergency CS consisted of approximately one-third of all CSs in Japan.^17^ Women undergoing emergency CS have been reported to have a higher risk of PPD than those with elective CS (OR 1.45; 95% CI, 1.04–2.01 vs OR 1.34; 95% CI, 0.94–1.92 in a previous study of 3,888 Swedish women,^47^ and OR 1.53; 95% CI, 1.02–2.31 vs OR 0.99; 95% CI, 0.56–1.75^11^ in a previous study of 4,941 Dutch women), so the investigation of overall CS and PPD could blur the real associations with emergency CS. Second, our outcome, PPD, was measured using self-reported EPDS scores and was not diagnosed by psychiatrists. However, the EPDS is commonly used in research and is a well-validated reliable measurement; therefore, the impact of misclassification is expected to be small. Third, residual confounding could have occurred from unmeasured confounding variables, such as the mother’s anxiety or fear of delivery and general fatigue.

In conclusion, women who underwent CS had a marginal association with PPD at 1 month after childbirth, but that association did not persist at 6 months. Women who had antenatal psychological distress and underwent CS may be regarded as targets for monitoring of PPD in the early stages after childbirth.
